# Sagittal spinal alignment and segmental mobility in collegiate baseball players with low back pain: A cross-sectional study

**DOI:** 10.1186/s12891-026-09530-5

**Published:** 2026-01-26

**Authors:** Takeshi Morifuji, Hidetoshi Nakao, Yasushi Kurihara, Hironori Ohsugi, Katsuyuki Morishita, Taizan Fukaya, Yahiko Takeuchi

**Affiliations:** Department of Physical Therapy, Faculty of Social Work Studies, Josai International University, 1 Gumyo, Togane-shi, Chiba 283-8555 Japan

**Keywords:** Baseball, Isometric trunk muscle strength, Low back pain, Segmental mobility, Spinal alignment

## Abstract

**Background:**

Low back pain is common among baseball players and may impair their performance and increase the risk of absence from competition. Abnormalities in the sagittal alignment of the spine and reduced trunk function have been suggested as risk factors, but evidence in collegiate players with skeletal maturity remains limited. This study investigated differences in sagittal alignment of the spine, segmental mobility, trunk flexibility, and isometric trunk muscle strength between collegiate baseball players with and without low back pain.

**Methods:**

This cross-sectional study included collegiate baseball players recruited from our university in July 2024. Players were classified into a low back pain (LBP) group and a control group based on the presence or absence of current low back pain (numeric rating scale ≥ 3). Spinal alignment was assessed in terms of segmental alignment, thoracic kyphosis, lumbar lordosis, and sacral inclination. Spinal mobility was evaluated through segmental motion, total thoracic and lumbar ranges of motion, and changes in sacral inclination during flexion and extension. Additional measures included trunk flexion (using the standing forward bend test), trunk extension (using the prone trunk extension test), and isometric trunk muscle strength (using a handheld dynamometer). Group comparisons were performed using the Mann–Whitney U test. Additionally, logistic regression analysis was conducted to identify independent factors associated with low back pain.

**Results:**

A total of 68 collegiate baseball players were analyzed (LBP: *n* = 35; Con: *n* = 33). The LBP group showed a significantly smaller lordosis angle at L1/2 compared with controls (–2.0° [–3.0 to − 1.0] vs. − 3.0° [–5.0 to − 1.0], *p* = 0.042). For segmental mobility, flexion ROM at L5/S1 was significantly greater in the LBP group (5.0° [2.0–8.0] vs. 3.0° [1.0–5.0], *p* = 0.030). No significant between-group differences were observed in other sagittal alignment parameters, global spinal mobility, overall trunk flexibility, or isometric trunk muscle strength (all *p* > 0.05). Logistic regression identified greater kyphotic alignment at L1/2 (OR = 1.263, 95% CI: 1.003–1.592, *p* = 0.047) and greater flexion ROM at L5/S1 (OR = 1.152, 95% CI: 1.009–1.315, *p* = 0.036) as independent predictors of low back pain.

**Conclusion:**

The findings indicate that alterations at the thoracolumbar junction (L1/2) and increased flexion mobility at the lumbosacral junction (L5/S1) are independently associated with low back pain in collegiate baseball players. These findings highlight the relevance of evaluating localized sagittal spinal mechanics, particularly at the thoracolumbar and lumbosacral junctions, to better understand low back pain in collegiate baseball players.

## Background

Low back pain (LBP) is a common musculoskeletal condition among baseball players. A review by Wasser et al. reported that the prevalence of LBP in baseball players ranges from 3% to 15% [1]. A study of Japanese professional baseball players reported that low back pain was attributable to lumbar disc herniation in 44%, discogenic pain in 22%, spondylolysis in 19%, facet joint arthritis in 6%, discal cyst in 3%, and nonspecific LBP in 6% [2]. Such conditions can reduce opportunities to participate in competitions and, in some cases, surgical intervention may be required; these conditions thus impose substantial disadvantages on players. Camp et al. reported that 12% of cases on the disabled list in Major League Baseball were attributable to back and core injuries, emphasizing the importance of trunk stability and spine control for maintaining athletic performance and preventing injuries.

In this context, physical functions such as trunk strength and flexibility have been recognized as modifiable risk factors that may contribute to the onset or recurrence of LBP [3]. Indeed, studies involving athletes have suggested that impairments in trunk function represent potential risk factors for LBP, with particular emphasis placed on reduced ranges of motion (ROMs) for lumbar flexion and extension as predictors of future onset [4]. In baseball players, however, most previous studies have focused on adolescents or high school athletes, possibly because the incidence of conditions such as spondylolysis is particularly high in this age group. For example, studies involving high school baseball players have demonstrated a significant association between LBP during lumbar extension and reduced core stability [5], and have also identified limited hamstring flexibility as a risk factor for LBP [6]. In addition, reduced quadriceps femoris flexibility has been associated with LBP in young baseball players [7], potentially by restriction of pelvic motion and increased lumbar loading. These findings provide valuable information about risk factors during growth and development, but little is known about risk factors for collegiate players. Collegiate athletes are generally considered to be near skeletal maturity, whereas younger athletes are still undergoing spinal growth and structural development. Therefore, the factors associated with LBP in collegiate athletes may differ from those observed in developmentally immature athletes. To clarify this distinction, it is important to consider how spinal structures change with maturation. Although spinal development progresses throughout childhood and adolescence, several lines of evidence indicate that key structural and biochemical characteristics of the spine begin to transition toward an adult-like pattern by late adolescence. Biochemical analyses of human intervertebral discs have demonstrated that water content and glycosaminoglycan concentration—highest in early childhood—begin to decline during late adolescence, with individuals aged 15–25 years already exhibiting substantially lower values than children aged 2–5 years [8]. Similarly, studies of spinal and pelvic alignment have reported that parameters such as lumbar lordosis, thoracic kyphosis, pelvic tilt, and sacral slope approach adult values and show relative stabilization around 16–18 years of age [9, 10]. These findings suggest that collegiate athletes aged 18 years and older can reasonably be considered to have reached a near-mature sagittal alignment profile.

Sagittal spinal alignment has been increasingly recognized as an important factor in the development of LBP in athletes. Evidence from young elite cross-country skiers has shown that athletes with greater overall sagittal spinal curvature—characterized by larger combined thoracic kyphosis and lumbar lordosis angles—have a higher prevalence of LBP [11]. In young baseball players, increased lumbar lordosis has been identified as a risk factor for spondylolysis [12, 13], suggesting that extension-biased sagittal alignment may predispose athletes to lumbar stress injuries. Additionally, specific sagittal morphotypes—such as functional hyperkyphosis and structured hyperlordosis—have been associated with recurrent LBP in competitive athletes [14].

Beyond static alignment, mobility-related factors may also influence spinal loading. Limited hamstring extensibility has been shown to restrict anterior pelvic tilt and reduce lumbar lordosis during trunk flexion, and this altered lumbopelvic movement pattern has been associated with recurrent LBP in team sport athletes [15]. Moreover, dynamic imaging studies in the general population with LBP have demonstrated excessive segmental motion at the lower lumbar spine [16]. Taken together, these findings indicate that abnormalities in sagittal alignment, lumbopelvic mobility, and segmental spinal motion may all contribute to lumbar loading and pain. These data highlight the need to evaluate not only overall spinal alignment and global spinal mobility, but also segmental spinal mobility, to better understand LBP mechanisms in athletic populations.

The Spinal Mouse^®^ system (Idiag AG, Zürich, Switzerland) has been used in studies on athletes as a noninvasive device for evaluating—without exposure to radiation—alignment and flexion–extension mobility of the spine, as well as intersegmental ROM [17–19]. Previous studies have demonstrated acceptable validity and good reproducibility of this device, particularly in the sagittal plane, supporting its suitability for field-based assessment of spinal function [20–22]. Although trunk rotation plays an important role in baseball-specific movements, particularly during pitching and batting, and has been shown to contribute to lumbar stress, flexion–extension movement of the spine is also considered a critical component of lumbar loading during athletic activities [1]. Previous studies in baseball players—primarily conducted in adolescent and growing athletes—have suggested that sagittal spinal alignment influences lumbar stress and injury risk. In addition, research in athletes has indicated that flexion–extension mobility may affect lumbar loading, while studies in individuals with low back pain have demonstrated excessive segmental motion at the lower lumbar spine. Collectively, these findings suggest that abnormalities in sagittal alignment, flexion–extension mobility, and segmental spinal motion may contribute to lumbar loading and pain. Thus, evaluating sagittal-plane spinal alignment, flexion–extension movement, and segmental spinal mobility is essential for understanding factors associated with low back pain in collegiate baseball players. Therefore, the purposes of this study were twofold: (1) to clarify differences in sagittal spinal alignment, flexion and extension mobility of the spine, and intersegmental ROM between collegiate baseball players with and without LBP using the Spinal Mouse^®^; and (2) to evaluate sagittal-plane–related trunk function measures—including trunk flexibility and trunk muscle strength—to identify physical characteristics associated with LBP.

Based on the biomechanical characteristics of baseball, which requires repetitive trunk extension [1], we expected that collegiate baseball players with LBP would exhibit increased lumbar lordosis, reflecting extension-based loading patterns and potential residual changes from previous lumbar stress injuries. Previous studies have shown that athletes exhibit larger sacrohorizontal angles than nonathletes, which is associated with increased lumbar lordosis [23]. Greater lumbar lordosis increases posterior compressive and shear forces at the lower lumbar spine, which may result in greater mechanical loading at these segments [24]. Furthermore, the pitching motion includes trunk extension during the arm-cocking phases and forward trunk inclination accompanied by spinal flexion during the follow-through [1, 25], and certain fielding situations, such as ground-ball handling, are generally thought to involve forward trunk inclination. As noted above, previous studies—primarily involving adolescent baseball players—have reported that players with LBP exhibit reduced flexibility of the hamstring and quadriceps femoris muscles. Reduced flexibility of these lower extremity muscles may influence pelvic motion during sagittal-plane trunk movement. In particular, limited hamstring flexibility has been shown to alter lumbopelvic rhythm, resulting in increased reliance on lumbar flexion during forward bending [26]. Based on these biomechanical considerations, it is plausible that baseball players with LBP may exhibit greater lumbar segmental motion during sagittal-plane activities. Accordingly, we hypothesized that players with LBP would show increased segmental mobility in both extension and flexion at the lower lumbar spine and the lumbosacral junction, which are subjected to relatively greater mechanical demands during sagittal-plane trunk motion.

## Methods

### Study design and ethical considerations

This study was a cross-sectional investigation conducted in July 2024. Ethical approval was obtained from the institutional review board of our university (approval number 05P24025). All participants were informed of the study procedures both verbally and in writing, and written informed consent was obtained from each participant prior to enrollment.

### Participants

The participants were male collegiate baseball players aged 18 years or older who belonged to the university hardball baseball team. Only male players were included because the university team consists exclusively of men, and the study aimed to investigate characteristics specific to this athletic population. Players with LBP were defined as those who reported a numeric rating scale (NRS) score of ≥ 3. Based on previous studies, an NRS score of ≥ 3 was considered to indicate pain levels that interfere with daily life and sports activities, and was therefore adopted as the inclusion criterion for the LBP group [27–29]. Players without LBP were allocated to the control (Con) group if they had an NRS score of 0 and reported no history of organic spine disorders such as lumbar spondylolysis or lumbar disc herniation. Information on the presence or absence of these disorders was obtained through a brief self-reported medical history form. The inclusion criteria did not specify a minimum duration of low back pain. Instead, athletes who were participating in baseball activities at the time of data collection were eligible; thus, players with severe acute inflammatory symptoms that could markedly affect spinal motion were unlikely to be included in the study population. All measurements were conducted in the laboratory of the Department of Physical Therapy at our university.

### Sample size calculation

The required sample size was calculated using G*Power software (version 3.1.9.6; Heinrich Heine Universität Düsseldorf, Germany) [30]. Based on a two-sample t-test (equivalent to the Mann–Whitney U test) for detecting differences in mean values between two groups, the effect size (Cohen’s d) was set at 0.8, the significance level (α) at 0.05, and the statistical power (1 − β) at 0.80. The analysis indicated that at least 26 participants per group (in total, 52) were required. The effect size was determined with reference to previous studies that used the Spinal Mouse^®^ to examine group differences in spinal alignment and mobility [31].

### Spinal alignment and mobility

**Fig. 1:**
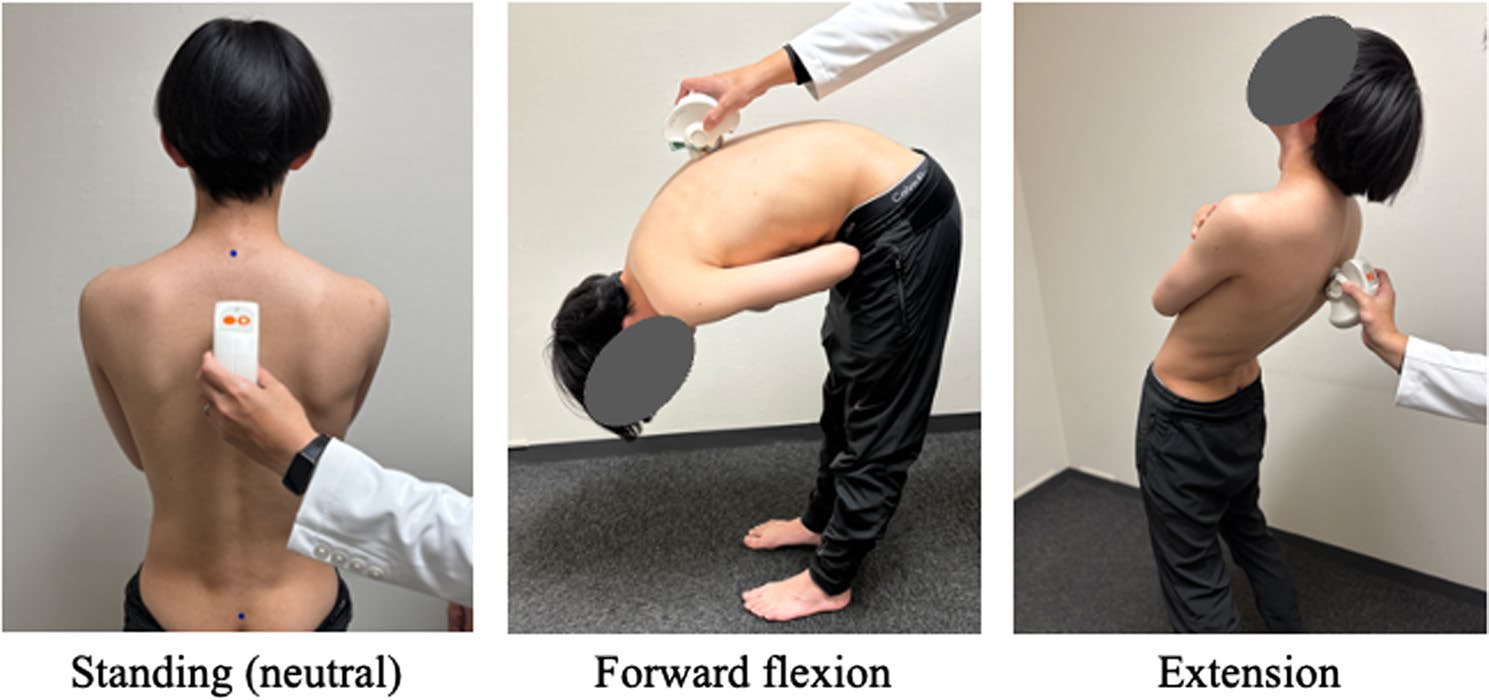
Measurement procedures using the Spinal Mouse^®^ The figure illustrates the three postures used to assess sagittal spinal alignment and segmental mobility with the Spinal Mouse^®^ system: neutral standing, maximal trunk flexion, and maximal trunk extension. Measurements were obtained by manually guiding the device along the spinous processes from C7 to S3 according to the manufacturer’s standardized protocol

Sagittal alignment and mobility of the spine were evaluated using the Spinal Mouse^®^ (Fig. 1). This device consists of a handheld, computer-assisted, mouse-shaped instrument with rolling wheels, and it is manually guided along the spinous processes to record curvature and mobility of the spine. All measurements were performed by one experienced physical therapist who was blinded to the study purpose.

The procedures followed standardized protocols described in previous reliability studies of the Spinal Mouse^®^ [21]. Before data collection, anatomical landmarks (C7 and S3) were palpated and marked on the skin to ensure accurate placement of the device. During each measurement, the device was guided along the spinous processes at a controlled, steady speed, as recommended to minimize measurement variability.

Previous studies have demonstrated acceptable measurement properties for the Spinal Mouse^®^, supporting its use as a reliable instrument for assessing sagittal spinal alignment and mobility [20–22]. In particular, Mannion et al. reported between-day ICC values ranging from 0.67 to 0.92 for global sagittal alignment and ranges of motion, with mean SEM values of approximately 3°. For segmental flexion (T1–2 to L5–S1), ICC values averaged around 0.6 and SEM values were approximately 2° [21]. All spinal alignment and mobility measurements were performed three times, and the mean values of the three trials were used for analysis. In this study, flexion and extension mobility were calculated as the change in angle from the upright static alignment to the maximum flexion or extension posture, rather than as absolute angle values.

### Spinal alignment

Thoracic kyphosis, lumbar lordosis, sacral inclination, and intersegmental angles from Th1/2 to L5/S1 were measured with the participant in a static standing position. Intersegmental angles were expressed as positive values for kyphosis (posterior convexity) and negative values for lordosis (anterior convexity). Sacral inclination was defined as the angle between the line connecting S1 to S3 and the vertical line; positive values indicated anterior tilt and negative values indicated posterior tilt. Participants stood barefoot with the upper body exposed, arms folded across the chest, feet a shoulder width apart, gaze maintained at eye level using a fixed reference point in front of them.

### Spinal mobility

Spinal mobility measurements were performed in a standing position, with participants maintaining the same posture as used during the static alignment assessment. Before the actual measurements, participants practiced the flexion and extension movements once in the same arm-folded posture, while keeping their knees fully extended, to familiarize themselves with the procedure. They then performed maximum voluntary spinal flexion and extension while maintaining the same arm-folded, knees-extended posture used during the practice trial. During both flexion and extension, care was taken to prevent knee flexion. Measurements included total thoracic and total lumbar ROM, the change in sacral inclination angle during flexion and extension, and intersegmental ROM from Th1/2 to L5/S1.

### Trunk function

Because the primary objective of this study was to examine sagittal-plane spinal alignment and segmental mobility using the Spinal Mouse^®^, the assessment of physical function was limited to measures directly related to sagittal-plane motion. Accordingly, trunk flexion/extension flexibility and isometric strength of the trunk flexor and extensor muscles were selected, as these parameters contribute to sagittal spinal loading and lumbar function in athletes.

Deep core stabilizers and three-dimensional trunk control—such as activation of the transversus abdominis or the internal and external oblique muscles—were not directly evaluated, as their assessment requires different measurement techniques (e.g., ultrasound imaging or electromyography), which were beyond the scope of this sagittal-plane–focused study.

Flexibility assessments are widely standardized in sports science, and established protocols for evaluating range of motion in athletic populations have been comprehensively summarized in previous literature [26].

### Standing trunk flexion

Flexibility in trunk flexion was measured using a digital standing trunk flexion meter (T.K.K. 5111; Takei Scientific Instruments Co., Ltd., Niigata, Japan). The test was conducted according to previously validated protocols [32]. Participants stood barefoot on the measurement platform with their feet a shoulder width apart and knees fully extended, then flexed the trunk slowly without bouncing, pressing the sliding marker downward with their fingertips until maximum flexion was reached. The value was recorded after the participant had held the end position for a few seconds. To ensure reliability, one physical therapy student consistently conducted all measurements.

### Prone trunk extension

 Trunk extension flexibility was assessed using an analog extension meter (Extension-A, T.K.K. 5004; Takei Scientific Instruments Co., Ltd.). Participants lay prone, with their arms alongside the trunk, and were instructed to slowly extend the trunk without bouncing. At maximum extension, the vertical distance from the chin to the floor was recorded. The prone trunk extension test has previously been reported to have high reliability for assessing lumbar extension mobility [33]. Two examiners, both physical therapy students, conducted the measurements: one fixed the participant’s feet, while the other recorded the maximum value using the extension meter. This test was also performed once, and the single recorded value was used for analysis .

### Trunk muscle strength

Isometric trunk flexor and extensor strength was measured using a handheld dynamometer (mobieZ, MT-201; SAKAI Medical Co., Ltd., Tokyo, Japan). Previous studies on adult populations have reported that handheld dynamometry has shown good reliability for measuring isometric trunk muscle strength [34]. For trunk flexor strength, participants were positioned in a supine position with their hips and knees flexed. The upper limbs were placed alongside the trunk. The sensor pad was positioned at the xiphoid process and secured with a fixation belt. For trunk extensor strength, participants were placed in a prone position with their knees extended, and the distal lower legs fixed. The upper limbs were placed alongside the trunk. The sensor pad was applied at the midpoint between the inferior angles of the scapulae and secured with a fixation belt. All measurements were performed on a treatment bed, and the fixation belts were secured directly to the bed to ensure adequate stabilization and prevent compensatory movements. For both tests, participants then performed a 5-second isometric contraction and were instructed to reach maximal effort within 3 s and maintain it isometrically. A single maximal trial was adopted to minimize testing burden, and previous work has shown high single-measurement reliability for handheld dynamometry in trunk strength assessment [34]. The peak forces (kgf) obtained during these single trials were adopted as the representative values. Torque was calculated by multiplying the force value (kgf) by the length of the lever arm (m) and then dividing this by body mass (kg) to obtain normalized values (kg·m/kg). The length of the lever arm was defined as the distance from the xiphoid process to the midpoint of the anterior superior iliac spines for flexion, and from the inferior angles of the scapulae to the upper margin of the sacrum for extension. One experienced physical therapist conducted all measurements.

### Data recording and processing

Measurement data from the Spinal Mouse^®^ system were automatically recorded and stored digitally using the dedicated software. Data from other physical tests—including standing trunk flexion, prone trunk extension, and trunk muscle strength—were recorded manually on standardized paper data sheets and subsequently digitized for analysis. Each participant was assigned a unique identification code, and all personal information was anonymized before data processing to ensure confidentiality.

### Statistical analysis

Statistical analyses were performed using IBM SPSS Statistics for Windows version 27 (IBM Corp., Armonk, NY, USA). The normality of the data was assessed using the Kolmogorov–Smirnov test. As most variables were non-normally distributed, descriptive statistics are presented as median and interquartile range (IQR).

Between-group comparisons (LBP vs. Con) were performed using the Mann–Whitney U test. Effect sizes (r) were calculated for each Mann–Whitney U test using the formula r = Z/√n.

The standard error of measurement (SEM) for each spinal parameter was calculated using the formula: SEM = SD × √(1 − ICC(1,3)). This approach is commonly used when repeated-measure ICC(1,3) values are available and reflects the absolute measurement error independent of sample size.

Binary logistic regression was planned to identify independent factors associated with low back pain. When significant differences were observed in the initial between-group comparisons, those variables were considered as candidate predictors for the multivariable logistic regression analysis. BMI was additionally included as a covariate to account for the potential influence of body size on spinal parameters and low back pain. A forward stepwise likelihood ratio method was applied. Model fit was assessed using the Hosmer–Lemeshow test, and odds ratios (ORs) with 95% confidence intervals (CIs) were calculated.

The significance level was set at *p* < 0.05 for all analyses. No adjustment for multiple comparisons was applied because the study was exploratory, and over-correction may increase Type II errors. Effect sizes were reported to aid interpretation.

## Results

**Fig. 2:**
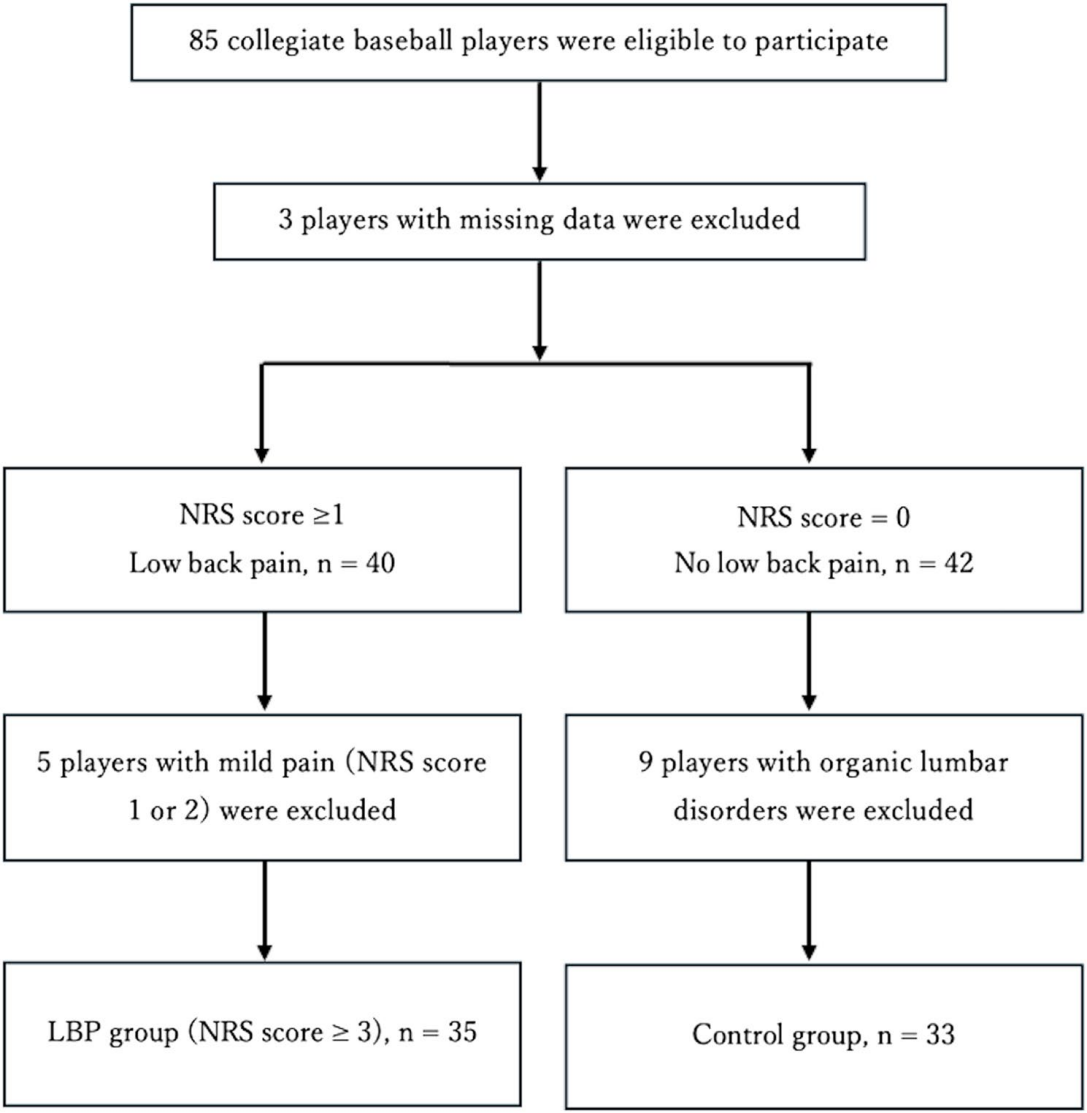
Flowchart showing participant selection. Abbreviations: *LBP* low back pain, *NRS* numeric rating scale

A total of 85 collegiate baseball players participated in this study. Three players with missing data were excluded. Of the 40 players who reported having LBP (an NRS score of ≥ 1), 5 with mild pain (an NRS score of 1 or 2) were excluded. The LBP group therefore consisted of 35 players with an NRS score of ≥ 3, including 3 players who self-reported a history of lumbar disc herniation and 9 who self-reported a history of lumbar spondylolysis. Of the 42 players with no LBP (an NRS score of 0), 9 had a history of organic spinal disorders (2 with lumbar disc herniation and 7 with spondylolysis), and these players were excluded; thus, the Con group comprised 33 participants (Fig. 2). None of the remaining players in the Con group reported a previous medical diagnosis of nonspecific low back pain based on the self-reported medical history. Among the 35 players in the LBP group, two reported symptom onset within the past 7 days and four within the past month. For players reporting symptoms beyond one month, however, the information was often vague or missing. Therefore, it was not feasible to summarize pain duration for the LBP group. These characteristics of the sample should be considered when interpreting the results.

The baseline characteristics and baseball training experience of the participants are shown in Table 1. No significant between-group differences were observed in age, height, body mass, body mass index, or baseball training experience. Additionally, the distribution of playing positions was not balanced between categories, with particularly small numbers of catchers in each group. Specifically, the Con group included 13 pitchers, 1 catcher, 11 infielders, and 8 outfielders, whereas the LBP group included 15 pitchers, 4 catchers, 8 infielders, and 8 outfielders. Because several tactical positions had inadequate sample sizes, these variables were not used for statistical comparisons and were therefore described only to characterize the sample.

**Table 1:** Baseline characteristics and baseball training experience of the two groups

Variable	Con group	LBP group	*p* value
Age, years	19.6 ± 1.2 (18–22)	19.8 ± 1.3 (18–22)	0.714
Height, cm	173.9 ± 5.3 (166.5–196.0)	173.0 ± 4.9 (164.3–183.5)	0.454
Weight, kg	71.4 ± 7.5 (59.7–97.0)	71.5 ± 9.1 (53.9–90.0)	0.873
Body mass index, kg/m^2^	23.5 ± 1.7 (20.1–26.9)	23.8 ± 2.4 (19.0–28.8)	0.619
Baseball training experience, years	12.5 ± 2.0 (8–16)	12.7 ± 2.3 (9–18)	0.586

Values are presented as mean ± standard deviation (min–max)

Abbreviations: *Con*, control, *LBP* low back pain

Regarding sagittal alignment of the spine, participants in the LBP group exhibited significantly smaller angles of lordosis at L1/2 than those in the Con group (p < 0.05; Table 2). No significant differences were found in other intervertebral angles of alignment, thoracic kyphosis, lumbar lordosis, or sacral inclination. For segmental spinal flexion ROM, the LBP group showed a significantly greater flexion ROM at the L5/S1 level than the Con group (p < 0.05; Table 3). No significant differences were observed in the ROMs for flexion at the other intervertebral levels, ROMs for total thoracic or total lumbar flexion, or the change in sacral inclination angle during flexion (Table 3)

**Table 2:** Comparison of sagittal spinal alignment parameters between the two groups

	Con	LBP	*p* value	Effect sizes
Th1/2, °	8.0 (4.5–12.5)	8.0 (5.0–11.0)	0.749	0.039
Th2/3, °	6.0 (3.5–8.0)	6.0 (4.0–10.0)	0.294	0.127
Th3/4, °	3.0 (1.0–4.0)	3.0 (1.0–5.0)	0.814	0.028
Th4/5, °	3.0 (0.5–4.0)	3.0 (0.0–3.0)	0.376	0.107
Th5/6, °	3.0 (2.0–4.0)	2.0 (1.0–4.0)	0.123	0.187
Th6/7, °	3.0 (2.0–6.0)	3.0 (2.0–4.0)	0.480	0.086
Th7/8, °	6.0 (4.0–8.5)	4.0 (2.0–7.0)	0.175	0.164
Th8/9, °	6.0 (4.0–8.0)	7.0 (4.0–8.0)	0.377	0.107
Th9/10, °	2.0 (–0.5–6.0)	5.0 (2.0–6.0)	0.068	0.221
Th10/11, °	1.0 (–1.0–3.5)	1.0 (–1.0–4.0)	0.549	0.073
Th11/12, °	–1.0 (–2.5–0.0)	0.0 (–2.0–1.0)	0.278	0.132
Th12/L1, °	–2.0 (–3.0–1.0)	–1.0 (–2.0–0.0)	0.257	0.138
L1/2, °	–3.0 (–5.0 – − 1.0)	–2.0 (–3.0 – − 1.0)	0.042*	0.247
L2/3, °	–4.0 (–6.0 – − 2.5)	–5.0 (–6.0 – − 3.0)	0.244	0.141
L3/4, °	–6.0 (–7.5 – − 3.5)	–6.0 (–8.0 – − 4.0)	0.635	0.058
L4/5, °	–5.0 (–8.0 – − 4.0)	–6.0 (–8.0 – − 3.0)	0.892	0.016
L5/S1, °	–5.0 (–6.0 – − 2.0)	–5.0 (–7.0 – − 2.0)	0.747	0.039
Thoracic kyphosis angle, °	39.0 (36.0–48.0)	43.0 (37–47.0)	0.393	0.104
Lumbar lordosis angle, °	–25.0 (–28.0 – − 21.0)	–23.0 (–28.0 – − 20.0)	0.731	0.042
Sacral inclination, °	12.0 (10.0–16.0)	11.0 (9.0–15.0)	0.457	0.090

Values are presented as median (interquartile range)

*p* < 0.05 was considered statistically significant

Abbreviations: *Con* control, *LBP* low back pain

**Table 3:** Comparison of spinal flexion range-of-motion parameters between the two groups

	Con	LBP	*p* value	Effect sizes
Th1/2, °	–4.0 (–8.0 – − 0.5)	–2.0 (–6.0–0.0)	0.434	0.095
Th2/3, °	–2.0 (–4.0–1.0)	–1.0 (–6.0–2.0)	0.907	0.014
Th3/4, °	1.0 (–1.5–3.5)	2.0 (0.0–5.0)	0.171	0.166
Th4/5, °	1.0 (–1.0–3.0)	2.0 (0.0–4.0)	0.254	0.138
Th5/6, °	0.0 (–1.5–2.0)	1.0 (0.0–3.0)	0.111	0.193
Th6/7, °	–1.0 (–3.0–1.5)	–1.0 (–2.0–1.0)	0.848	0.023
Th7/8, °	–1.0 (–4.5–0.0)	–1.0 (–4.0–1.0)	0.416	0.099
Th8/9, °	1.0 (–2.0–4.0)	0.0 (–3.0–2.0)	0.148	0.175
Th9/10, °	5.0 (1.5–9.0)	3.0 (1.0–6.0)	0.091	0.205
Th10/11, °	5.0 (3.0–7.5)	7.0 (4.0–10.0)	0.158	0.171
Th11/12, °	5.0 (4.0–8.0)	5.0 (2.0–8.0)	0.583	0.067
Th12/L1, °	5.0 (2.0–6.5)	4.0 (2.0–7.0)	0.873	0.019
L1/2, °	9.0 (6.0–10.5)	8.0 (4.0–10.0)	0.075	0.216
L2/3, °	12.0 (10.0–14.5)	13.0 (10.0–15.0)	0.456	0.090
L3/4, °	15.0 (12.0 − 19.0)	16.0 (13.0–18.0)	0.744	0.040
L4/5, °	12.0 (9.0–15.0)	13.0 (6.0–18.0)	0.907	0.014
L5/S1, °	3.0 (1.0–5.0)	5.0 (2.0–8.0)	0.030*	0.264
ROM of total thoracic flexion, °	14.0 (4.0–19.0)	13.0 (5.0–22.0)	0.560	0.010
ROM of total lumbar flexion, °	55.0 (51.0–63.5)	57.0 (52.0–65.0)	0.731	0.045
Change in sacral inclination angle, °	50.0 (40.0–60.5)	47.0 (40.0–54.0)	0.323	0.063

Values are presented as median (interquartile range)

p< 0.05 was considered statistically significant

Abbreviations: *Con* control, *LBP* low back pain, *ROM* range of motion

No significant differences were found in the ROMs for extension of the spine at all intervertebral levels, total thoracic extension, or total lumbar extension, or the change in sacral inclination angle during extension (Table 4). No significant between-group differences in the standing forward bend or prone trunk extension test findings were noted (Table 5). Trunk flexion and extension strength did not differ significantly between the groups (Table 5).

**Table 4:** Comparison of spinal extension range-of-motion parameters between the two groups

	Con	LBP	*p* value	Effect sizes
Th1/2, °	–5.0 (–7.0 – − 1.0)	–3.0 (–5.0–0.0)	0.161	0.034
Th2/3, °	–1.0 (–3.5–2.0)	–3.0 (–5.0–0.0)	0.073	0.158
Th3/4, °	0.0 (–2.0–1.0)	–1.0 (–2.0–1.0)	0.916	0.189
Th4/5, °	0.0 (–3.5–2.0)	0.0 (–2.0–1.0)	0.734	0.123
Th5/6, °	–1.0 (–3.0–0.0)	0.0 (–2.0–1.0)	0.083	0.006
Th6/7, °	–1.0 (–2.0–1.0)	0.0 (–2.0–2.0)	0.512	0.073
Th7/8, °	–2.0 (–4.5–1.0)	–1.0 (–4.0–2.0)	0.328	0.112
Th8/9, °	–1.0 (–3.5–1.5)	–2.0 (–4.0–0.0)	0.264	0.073
Th9/10, °	0 (–3.0–5.0)	1.0 (–3.0–3.0)	0.438	0.067
Th10/11, °	1.0 (–3.0–4.0)	1.0 (0.0–4.0)	0.346	0.068
Th11/12, °	0.0 (–5.0–4.5)	–1.0 (–3.0–4.0)	0.792	0.105
Th12/L1, °	–1.0 (–4.5–2.5)	–4.0 (–5.0–0.0)	0.143	0.201
L1/2, °	–4.0 (–5.5 – − 1.0)	–4.0 (–6.0–0.0)	0.985	0.158
L2/3, °	–3.0 (–8.0 – − 0.5)	–2.0 (–5.0–0.0)	0.289	0.124
L3/4, °	–4.0 (–8.0 – − 1.0)	–4.0 (–8.0–0.0)	0.951	0.036
L4/5, °	–7.0 (–11.5 – − 2.5)	–7.0 (–10.0 – − 2.0)	0.990	0.037
L5/S1, °	–5.0 (–8.0 – − 2.0)	–5.0 (–9.0 – − 2.0)	0.844	0.225
ROM of total thoracic extension, °	–4.0 (–19.5–4.0)	–9.0 (–18.0–3.0)	0.932	0.031
ROM of total lumbar extension, °	–26.0 (–31.0 – − 18.0)	–24.0 (–31.0 – − 18.0)	0.712	0.007
Change in sacral inclination angle, °	–18.0 (–23.0 – − 11.5)	–16.0 (–21.0 – − 10.0)	0.602	0.154

Values are presented as median (interquartile range)

p < 0.05 was considered statistically significant

Abbreviations: *Con* control, *LBP* low back pain, *ROM* range of motion

**Table 5:** Comparison of trunk flexibility and muscle strength between the two groups

Measurement	Con	LBP	*p* value	Effect sizes
Standing forward bend test, cm	6.2 (1.9–14.1)	6.2 (0.6–11.1)	0.394	0.112
Prone trunk extension test, cm	48.5 (43.3–52.0)	48.3 (43.4–53.1)	0.602	0.063
Trunk flexion strength, kg•m/kg	0.12 (0.10–0.13)	0.12 (0.10–0.14)	0.707	0.046
Trunk extension strength, kg•m/kg	0.19 (0.16–0.23)	0.19 (0.17–0.21)	0.616	0.061

Values are presented as median (interquartile range)

*p* < 0.05 was considered statistically significant

Abbreviations: *Con* control, *LBP* low back pain

The SEM values for segmental alignment and ROM measurements ranged from 0.6° to 1.4° for upright assessments, 0.9° to 2.1° for flexion assessments, and 1.1° to 3.2° for extension assessments (Table 6). These values indicate that the statistically significant differences observed at L1/2 and L5/S1 exceeded the corresponding SEMs, suggesting that the group differences were unlikely to be explained by measurement error alone.Binary logistic regression identified two significant independent predictors of low back pain. A greater kyphotic angle at L1/2 was a significant predictor (OR = 1.263, 95% CI: 1.003–1.592, p = 0.047). Greater flexion ROM at L5/S1 was also associated with increased odds of low back pain (OR = 1.152, 95% CI: 1.009–1.315, p = 0.036). BMI was not retained in the final model (p = 0.469).

**Table 6:** Intraclass correlation coefficients [ICC(1,3)], 95% confidence intervals, and standard error of measurement (SEM) for segmental sagittal alignment, flexion ROM, and extension ROM

	Sagittal spinal alignment			Flexion ROM			Extension ROM		
	ICC(1,3)	95% CI	SEM (°)	ICC(1,3)	95% CI	SEM (°)	ICC(1,3)	95% CI	SEM (°)
Th1/2	0.890	0.771–0.953	1.4	0.764	0.507–0.899	2.1	0.574	0.111–0.817	2.2
Th2/3	0.745	0.468–0.891	1.2	0.828	0.640–0.926	1.5	0.659	0.288–0.854	1.8
Th3/4	0.846	0.678–0.934	0.9	0.808	0.599–0.918	1.5	0.707	0.388–0.874	1.1
Th4/5	0.888	0.767–0.952	0.7	0.806	0.596–0.917	1.1	0.490	−0.063–0.782	1.3
Th5/6	0.795	0.573–0.912	0.8	0.830	0.645–0.927	1.2	−0.408	−1.937–0.396	1.3
Th6/7	0.927	0.849–0.969	0.9	0.913	0.819–0.963	1.1	0.646	0.261–0.848	1.3
Th7/8	0.943	0.881–0.975	0.8	0.751	0.480–0.893	1.2	0.761	0.502–0.898	1.4
Th8/9	0.940	0.874–0.974	0.9	0.973	0.943–0.988	0.9	0.832	0.650–0.928	1.6
Th9/10	0.871	0.731–0.945	1.0	0.843	0.673–0.933	1.5	0.591	0.147–0.825	2.1
Th10/11	0.878	0.740–0.949	0.9	0.818	0.620–0.922	1.3	0.729	0.436–0.884	2.1
Th11/12	0.872	0.732–0.945	0.8	0.830	0.645–0.927	1.2	0.803	0.589–0.915	2.1
Th12/L1	0.822	0.629–0.924	0.6	0.825	0.635–0.925	1.2	0.640	0.248–0.846	2.0
L1/2	0.818	0.620–0.922	0.8	0.805	0.593–0.916	1.1	0.773	0.526–0.903	1.8
L2/3	0.711	0.397–0.876	0.8	0.817	0.618–0.921	1.3	0.739	0.455–0.888	2.2
L3/4	0.866	0.721–0.943	0.6	0.888	0.765–0.952	1.1	0.509	–0.024–0.790	2.6
L4/5	0.930	0.853–0.970	0.8	0.808	0.600–0.918	1.6	0.330	–0.397–0.713	3.2
L5/S1	0.878	0.746–0.948	0.9	0.826	0.638–0.926	1.6	0.543	0.047–0.804	2.2
Thoracic kyphosis angle	0.225	–0.618–0.668	4.9	0.552	0.065–0.808	2.8	0.480	–0.085–0.777	6.6
Lumbar lordosis angle	0.964	0.924–0.984	1.0	0.781	0.542–0.906	6.5	0.765	0.509–0.899	4.7
Sacral inclination	0.968	0.932–0.986	1.5	0.987	0.973–0.994	1.4	0.821	0.626–0.923	6.3

Values are ICC(1,3) with 95% confidence intervals and standard error of measurement (SEM)

SEM values are expressed in degrees (°)

Abbreviations: *ROM* range of motion

## Discussion

This study aimed to clarify differences in sagittal spinal alignment, segmental mobility, trunk flexibility, and muscle strength between collegiate baseball players with and without LBP. The main findings were that the LBP group exhibited a significantly smaller angle of lordosis at L1/2 and a significantly greater ROM for flexion at L5/S1 than the Con group. By contrast, no significant between-group differences were observed in conventional indicators of trunk flexibility or strength. These findings suggest that segment-specific alterations in spinal alignment and mobility may be more closely associated with LBP in collegiate baseball players than global sagittal–plane–related trunk function. 

Previous studies have reported conflicting results regarding spinopelvic alignment in individuals with disorders of the lumbar spine. Endo et al. demonstrated that patients with lumbar disc herniation exhibited less lumbar lordosis and more vertical pelvic tilt, implicating spinopelvic malalignment in dysfunction of the spine [35]. Matsuzawa et al., however, reported that baseball players with spondylolysis showed more lumbar lordosis and anterior pelvic tilt [12]. In the present study, no between-group differences were observed in overall lumbar lordosis, possibly reflecting the inclusion of players with a history of both disc herniation and spondylolysis. Because these conditions are typically associated with decreased and increased lumbar lordosis, respectively, their coexistence may have attenuated group differences in global alignment. Furthermore, recent imaging research indicates that subclinical lumbar bone stress injuries can be present even in asymptomatic young athletes. Kountouris et al. reported that bone marrow oedema detectable on MRI often precedes symptomatic bone stress injury in junior fast bowlers [36]. Thus, some asymptomatic players in the control group may have had latent structural pathology that could influence lumbar curvature toward either increased or decreased lordosis, which could have further reduced between-group differences in global lumbar lordosis. Consequently, our original hypothesis predicting increased lumbar lordosis in the LBP group was not supported. 

Previous biomechanical studies have shown that repetitive trunk extension near the position of maximum external rotation during pitching imposes mechanical stress on the lumbar spine [37]. Prior studies have highlighted the thoracolumbar junction as a region particularly vulnerable to sagittal imbalance. Liu et al. reported that excessive kyphosis at the thoracolumbar junction contributes to global sagittal malalignment in patients with degenerative kyphosis [38], and Han et al. demonstrated that local kyphosis at this junction can progress with age even in healthy adults [39]. These findings suggest that the thoracolumbar junction is structurally predisposed to early postural changes, which may alter load transfer through the lumbar spine during dynamic movements. Building on this concept, the reduced L1/2 lordosis observed in the LBP group can be interpreted as a localized alteration at the thoracolumbar junction. Because L1/2 marks the beginning of the lumbar lordotic curve, diminished curvature at this level may change how extension-related mechanical stresses are absorbed and redistributed during pitching movements. Even when segmental mobility is preserved, a flatter upper lumbar alignment may reduce the ability of the thoracolumbar region to accommodate the demands of trunk extension, thereby shifting mechanical load to the lower lumbar segments. This alignment-based mechanism provides a plausible explanation for the association between reduced L1/2 lordosis and LBP in collegiate baseball players.

In the present study, global trunk flexibility was similar between the two groups, suggesting that traditional assessments of trunk flexibility alone may not adequately capture the presence of LBP in baseball players. In particular, the standing forward bend test may reflect hamstring flexibility rather than spinal function, which limits its use as the sole indicator of lumbar mobility. A systematic review by Moradi et al. suggested that restricted lumbar ROM may be associated with LBP in athletes [4], whereas a review by Victora Ruas and Vieira indicated that flexibility of the spine is not consistently related to LBP in the general population [40]. The present results align more closely with those of the latter study, indicating that the association between spinal mobility and LBP may vary according to population and sport-specific demands.Notably, in the present study we identified a significantly greater flexion ROM at L5/S1 in players with LBP, suggesting that segmental hypermobility at the lower lumbar spine is a potential contributor to chronic LBP. This finding aligns with the observations of Kulig et al., who used dynamic MRI to evaluate patients with nonspecific LBP; these researchers reported excessive motion at L4/5 and L5/S1 [16]. Similarly, Hershkovich et al. found that young adults with joint hypermobility exhibited a high prevalence of LBP, supporting the role of segmental instability as a risk factor [41]. Lao et al. used kinetic MRI to demonstrate that lumbar motion changes according to the degree of disc degeneration, with increased translational motion in mild to moderate degeneration [42]. In contrast, Basques et al. observed a decreased ROM for flexion–extension at multiple levels in the lumbar spine in individuals with LBP from the general population [43]. This apparent discrepancy may reflect differences in pathology, activity levels, and physical demands. Taken together, these findings suggest that lumbar segmental mobility can vary depending on structural characteristics and movement demands. To interpret why increased flexion mobility specifically emerged at L5/S1 in baseball players with LBP, it is necessary to consider the sport-specific demands placed on the lumbopelvic region. Pitching follow-through involves forward trunk flexion while rotational motion continues, imposing combined sagittal and transverse demands on the lumbar spine [1]. In contrast, many fielding actions—such as approaching ground balls—primarily involve forward trunk inclination. When anterior pelvic tilt is insufficient and pelvic contribution to forward trunk inclination is limited, lumbar flexion tends to shift distally, increasing motion demands at L5/S1. Experimental work has also shown that posterior pelvic tilt increases flexion stress at the lumbosacral junction, particularly at L5/S1 [44]. From this perspective, the greater flexion mobility observed at L5/S1 in the LBP group may reflect repeated compensatory recruitment of this segment during pitching and fielding movements requiring trunk flexion. 

Another important consideration when interpreting these findings is the potential influence of acute inflammatory processes. Acute inflammatory low back pain is typically associated with protective muscle activity and pain-avoidance behavior, leading to restricted spinal motion. In contrast, the present study demonstrated increased segmental flexion mobility at the L5/S1 level in players with LBP. This pattern suggests that the observed alterations are unlikely to be solely attributable to acute inflammatory processes and may instead reflect increased segmental loading at the lower lumbar spine. In addition, all participants were able to participate in baseball practice at the time of assessment, suggesting that severe acute inflammatory symptoms associated with marked restriction of spinal motion were unlikely in the study population. Nevertheless, because a small number of players reported symptom onset within the past 7 days or within the past 1 month, the influence of acute inflammatory processes cannot be completely excluded.

In addition to the biomechanical interpretation of these findings, it is also important to consider their statistical magnitude. Although the effect sizes were small—indicating limited magnitude and warranting cautious interpretation—it was also necessary to examine whether measurement precision could have influenced the observed differences. To ensure the robustness of these segmental findings, we compared the magnitude of the observed between-group differences with the standard error of measurement (SEM) derived from the reliability analysis of the Spinal Mouse®. Using the updated ICC (1,3) values from our dataset, the SEM for upright alignment at L1/2 was 0.8°, and the difference between the LBP and control groups was 1° based on median values (–3° vs –2°). For flexion ROM at L5/S1, the SEM was 1.6°, whereas the between-group differences were 2° in median values (3° vs 5°). Although these differences were small and close to the magnitude of the SEM, they exceeded measurement error, indicating that they represent true group-level differences rather than random variability. Nevertheless, their small absolute magnitude warrants cautious interpretation.

Furthermore, to clarify whether these segmental characteristics contributed independently to LBP, we additionally performed a logistic regression analysis. The results further strengthened our interpretation, identifying both reduced lordosis at L1/2 and increased flexion mobility at L5/S1 as independent factors associated with LBP. This indicates that these segmental characteristics contribute to LBP beyond the influence of overall body size (BMI), which was not retained in the final model. Together, these findings suggest that specific alterations at the thoracolumbar junction and lumbosacral region represent distinct mechanical signatures of LBP in collegiate baseball players, rather than generalized abnormalities in global alignment or trunk function. Although the effect sizes for L1/2 alignment and L5/S1 flexion mobility in the Mann–Whitney U tests were small, these variables also emerged as significant independent predictors in the multivariable logistic regression model. This analytical consistency reduces the likelihood that the univariate findings were due solely to Type 1 error and instead suggests that these segmental characteristics represent meaningful contributors to low back pain in collegiate baseball players.

By contrast, our hypothesis predicted increased segmental mobility at the lower lumbar spine in both flexion and extension; however, only flexion mobility was greater in the LBP group. Baseball players are repeatedly exposed to lumbar extension stresses during batting, fielding actions, and various throwing mechanics. These sport-specific demands suggest that increased extension mobility could theoretically occur in athletes with LBP. However, the measurement reliability for extension ROM at the lower lumbar spine was notably lower in our Spinal Mouse® analysis, particularly at L4/5 (ICC = 0.330) and, to a lesser extent, at L5/S1 (ICC = 0.543). This reduced reliability increases the likelihood of Type II error for extension measurements. Therefore, the absence of a significant between-group difference in extension ROM should be interpreted with caution, as it may reflect limitations in measurement precision rather than a true absence of extension-related mobility alterations.

When trunk muscle strength was evaluated, no significant between-group differences were observed in isometric flexion or extension strength. This may reflect that athletes generally maintain trunk strength above a certain threshold, making muscle weakness a less likely primary determinant of LBP. Previous studies have reported inconsistent findings: Cho et al. found that reduced trunk strength was associated with the severity of LBP in patients with chronic pain [45]. However, in a systematic review by Althobaiti et al., emphasis was placed on the association between trunk muscle weakness and LBP being inconsistent, with discrepancies across studies possibly being attributable to differences in assessment methods and participant characteristics [46]. Similarly, Paalanne et al. found no association between isometric strength of the trunk and LBP in young adults [47]. Instead, evidence recently reported by Hides et al. emphasized the importance of motor control and recruitment of the deep trunk muscle in maintaining spinal stability; this suggests that impaired motor control rather than absolute strength may underlie LBP [48]. In line with this, our findings of altered alignment and segmental mobility but preserved trunk strength imply that structural and functional instability, rather than strength deficits, play a more central role in LBP among collegiate baseball players. However, because this study focused on sagittal-plane mechanics, the trunk muscle assessments were limited to flexion/extension-related isometric strength. Therefore, the absence of between-group differences in isometric strength does not exclude the possibility of dysfunction in deep trunk stabilizers or motor control, which were beyond the scope of this study and may still contribute to LBP in this population.

This study had several limitations. First, the participants were drawn from a single collegiate baseball team, limiting generalizability to other sports or athletic populations. Second, in the present study, based on the biomechanical rationale described above, we primarily conceptualized segmental sagittal alignment and mobility as candidate risk factors for low back pain. However, because of the cross-sectional design, the observed differences in alignment and ROM should be interpreted as movement characteristics associated with LBP, rather than definitive evidence of causal direction. Furthermore, individuals with LBP often exhibit reduced spinal mobility as an adaptive response to pain. Therefore, alterations in ROM in athletes should be considered not only as potential risk factors for the development of LBP, but also as possible consequences of pain-related movement limitation. Third, although the Spinal Mouse® provides a convenient, noninvasive means of assessing spinal alignment and mobility, it relies on measurements taken at the surface of the skin and, unlike radiographic methods, it does not directly visualize bony or disc structures. Consequently, measurement error is unavoidable. In the present reliability analysis, most spinal segments demonstrated acceptable ICC(1,3) values; however, thoracic extension at Th5/6 showed notably low reliability. Importantly, this segment was not included in the variables showing between-group differences, and therefore does not affect the primary findings of this study. Nevertheless, the device offers practical utility as a screening tool in clinical and sports settings due to its noninvasive nature and ease of use. Fourth, the standing trunk flexion test reflects a combination of trunk flexion and hamstring flexibility [49]. Because movements at the spine and hamstrings contribute simultaneously to the final measurement, this test does not represent a pure assessment of trunk flexion ability. Nevertheless, it remains a practical and widely used field test due to its simplicity and feasibility. Fifth, trunk muscle assessments were limited to flexion/extension-related isometric strength, and deep trunk stabilizers or three-dimensional motor control were not evaluated. These factors may also influence LBP but were beyond the scope of this sagittal-plane–focused study. 

Although the presence of LBP may have influenced segmental mobility through pain-related guarding, this potential bias cannot fully explain the increased flexion ROM observed at L5/S1. This suggests that segmental hypermobility itself—rather than reduced mobility caused by pain—may be a contributing factor to LBP in this population. Finally, although hamstring extensibility and lower-limb flexibility have been shown to influence lumbopelvic alignment and sagittal spinal curves in athletes [15], and lower-limb ROM limitations can predict sagittal spinal misalignments in younger populations [50], this study did not assess lower-limb flexibility. Therefore, potential interactions between lower-limb flexibility and sagittal spinal alignment could not be examined. Future studies should incorporate lower-limb flexibility measures to more comprehensively evaluate contributors to LBP in collegiate baseball players. In addition, future research should compare the segmental sagittal spinal values observed in collegiate baseball players with normative sagittal morphotype classifications—such as those reported in the ISQUIOS programme—to better contextualize the present findings.

## Conclusions

This study clarified the importance of assessing spinal alignment and mobility at the segmental level in collegiate baseball players. In particular, segment-specific characteristics at the thoracolumbar and lumbosacral junctions may provide useful information for developing clinical screening and training strategies for athletes. Future studies should investigate whether interventions targeting these localized characteristics can help reduce the burden of low back pain in athletic populations.

## Data Availability

The datasets generated and/or analyzed during the current study are not publicly available due to privacy restrictions but are available from the corresponding author on reasonable request.
